# A Checklist to Improve Patient Safety in Interventional Radiology

**DOI:** 10.1007/s00270-012-0395-z

**Published:** 2012-05-05

**Authors:** Inge C. J. Koetser, Eefje N. de Vries, Otto M. van Delden, Susanne M. Smorenburg, Marja A. Boermeester, Krijn P. van Lienden

**Affiliations:** 1 Department of Interventional Radiology, Academic Medical Centre, Amsterdam, The Netherlands; 2 Department of Quality and Process Innovation, Academic Medical Centre, Amsterdam, The Netherlands; 3 Department of Surgery, Academic Medical Centre, Amsterdam, The Netherlands; 4 Meibergdreef 9, G1-229, 1105 AZ Amsterdam, The Netherlands

**Keywords:** Checklist, Interventional radiology, Patient safety

## Abstract

**Purpose:**

To develop a specific RADiological Patient Safety System (RADPASS) checklist for interventional radiology and to assess the effect of this checklist on health care processes of radiological interventions.

**Materials and Methods:**

On the basis of available literature and expert opinion, a prototype checklist was developed. The checklist was adapted on the basis of observation of daily practice in a tertiary referral centre and evaluation by users. To assess the effect of RADPASS, in a series of radiological interventions, all deviations from optimal care were registered before and after implementation of the checklist. In addition, the checklist and its use were evaluated by interviewing all users.

**Results:**

The RADPASS checklist has two parts: A (Planning and Preparation) and B (Procedure). The latter part comprises checks just before starting a procedure (B1) and checks concerning the postprocedural care immediately after completion of the procedure (B2). Two cohorts of, respectively, 94 and 101 radiological interventions were observed; the mean percentage of deviations of the optimal process per intervention decreased from 24 % before implementation to 5 % after implementation (*p* < 0.001). Postponements and cancellations of interventions decreased from 10 % before implementation to 0 % after implementation. Most users agreed that the checklist was user-friendly and increased patient safety awareness and efficiency.

**Conclusion:**

The first validated patient safety checklist for interventional radiology was developed. The use of the RADPASS checklist reduced deviations from the optimal process by three quarters and was associated with less procedure postponements.

## Introduction

A recent systematic review has shown that nearly one out of every ten patients admitted to a hospital will experience an adverse event [1]. Almost half of in-hospital adverse events are related to invasive procedures such as surgical procedures, endoscopy, or radiological interventions [1]. In addition to the inherent risk associated with invasive procedures, there are a number of other explanations for this disproportionate contribution. The presence of multiple patient transfer moments, the different specialties and types of personnel involved, and the complexity of the procedures may render these disciplines more prone to errors and adverse events [2, 3].

Interventional radiology (IR) is a fast-developing discipline with procedures and equipment getting more advanced and complicated by the day. Increasingly invasive procedures are being performed in a wide variety of patients, many of whom have not been evaluated by the interventional radiologist before the intervention. In IR, as in all medical disciplines, the need for improvements in quality and patient safety is increasingly being recognized [4–7]. Standard operating procedures are useful and important but do not contribute to a more systematic workflow; nor do they cover the entire pathway of an intervention.

The importance of safety checks has long been recognized in other areas, including aviation and other high-risk industries [8, 9]. In 2000, the Institute of Medicine recommended the implementation of verification processes, such as checklists, into medical practice to standardize processes and decrease reliance on human memory [10]. Recently, the World Health Organization has introduced a safety checklist in the operating room that reduces the rates of death and complications associated with surgery [11]. An even greater effect on mortality and complications in hospitals with high standard of health care quality is seen after implementation of the SURgical Patient Safety System (SURPASS) checklist that covers the entire surgical in-hospital pathway [12].

Because IR shares several features with surgery, a checklist may be equally effective to improve patient safety in IR [13]. Recently the Cardiovascular and Interventional Society of Europe published a checklist for IR [14]. This checklist was modified from the World Health Organization surgical safety checklist and the RADiological Patient Safety System (RADPASS) checklist. The RADPASS checklist is the subject and result of our study and it is the first validated safety checklist for the complete pathway of radiological interventions. The aim of this study was to design a specific checklist for IR, and to assess the effect of this checklist on health care processes of radiological interventions.

## Materials and Methods

Institutional review board (IRB) approval and informed consent were not deemed necessary in this observational study. Under Dutch law, the Medical Research Involving Human Subjects Act, IRB approval is required only when subjects are subjected to an intervention. Because this was an observational study where the effect of a quality improvement measure was studied (i.e., there was no additional risk or burden for subjects), IRB review was waived. The study meets all requirements regarding HIPAA compliance.

### Preimplementation Process

No standardized system for the preparation of IR procedures was operative before the implementation of the RADPASS checklist. Checking safety matrices (e.g., laboratory values, contrast allergies, medications) were left to individual initiative of the ward doctor in in-hospital patients, the referring doctor in outclinic patients, or the radiologist, which resulted in interpersonal variations in timing and method of checking safety items. Protocols were accessible, but they merely cover the procedure itself and not the complete pathway of interventions.

The tertiary referral center in which the study was conducted had no specific preprocedure clinic; however, patients in the short-stay clinic were routinely examined before the procedure. Patients admitted to the hospital were visited at the ward and given explanations about the procedure. The patient’s procedural preparation was performed by the referring physician.

### Checklist Development

The design of the RADPASS checklist was based on the structure of the previously described SURPASS checklist, a multidisciplinary checklist standardizing the entire in-hospital surgical pathway from admission until discharge [15] that reduces mortality by half and complications by a third [12]. For item selection of the RADPASS checklist, all available literature on errors, adverse events, and complications in IR, including various standards and guidelines, was consulted [4–7, 13, 16–21]. Using the information from these sources, a first set of checklist items was determined by an expert panel consisting of two experienced interventional radiologists (K.R.vL, O.M.vD) and a safety expert (S.M.S.). This first issue checklist was then tested in practice in a short trial period of 3 weeks by the two interventional radiologists of the expert panel. This period was subsequently evaluated and some modifications were made, leading to a prototype RADPASS checklist ready for broader testing.

### Before-and-After Study of the Checklist Effect

An observational before–after study was conducted in a tertiary referral center to assess the effect of the checklist on daily care processes. The broader scope of different types of IR procedures frequently performed (vascular/nonvascular, elective/semielective) were included. Patients undergoing neuro-interventional procedures and patients undergoing emergency procedures (e.g., embolization for trauma in hemodynamically unstable patient) were excluded from this study. Although the checklist is developed for all types of procedures, the emergency procedures were not suitable (because of a totally different workflow) for evaluation and further development of the checklist in this stage.

During real-time observations by an independent investigator of a series of randomly selected radiological interventions in a nonchecklist situation, all process deviations were scored on a structured observation form. Process deviations were defined as situations where an aspect of health care had not been executed correctly (e.g., “right side not marked” or “contrast allergy not checked”). A process deviation did not necessarily correspond to an adverse outcome, which denotes an actual unintended result for the patient that is both unwanted or negative and is related to medical management. Process deviations leading to rescheduling a patient were scored as a stopped-procedure event. Depending on the consequence, the event was defined as either a postponement (later the same day) or a cancellation (another day).

Observational data were used to further refine the prototype checklist. All process deviations were discussed within the expert panel, and any deviations that were not yet covered by the prototype checklist were added as items.

All interventional radiologists and technicians were then instructed on the use of the checklist, and the checklist was implemented in daily practice in all IR rooms. After a period of 6 months to allow for adequate implementation of the checklist, a similar series of observations was conducted. To prevent bias, participating interventional radiologists were not informed about the study design, were instructed as being involved in a quality improving project, and were unaware of the fact that effect evaluation was being performed. Fellows and residents also performed the checks and were supervised by a senior interventional radiologist, who was ultimately responsible for completing the checklist. In this way, it had an additional educational value, preparing the next-day IR patients.

Per procedure, the number and percentage of process deviations were calculated over the number of items characterizing an optimal process. Differences between the two cohorts were tested by the χ^2^ and Mann–Whitney *U*-test. All statistical analyses were two-tailed, and values of *p* < 0.05 were considered significant. The statistical analyses were completed by SPSS software, version 15.0 (SPSS, Chicago, IL).

### Evaluation by Users

After a 6-month period of use and after finishing all observations, structured evaluation interviews were conducted among all staff members, fellows, and technicians who had used the checklist. An independent investigator asked users about their personal experiences when using the list, suggestions for improvement, and their opinion whether the use of RADPASS improved patient safety (awareness) and efficiency. The interview consisted of 16 questions divided over three parts: usage of and satisfaction with the checklist, content of the checklist, and the effect of the checklist on the working process and patient safety. Questions were either multiple-answer possibilities, statements graded from 1 (completely agree) to 5 (completely disagree), or closed yes/no questions with a request for additional explanation. There was also room for suggestions and comments.

## Results

### Design of Optimized RADPASS Checklist

During the adaptation rounds, various modifications were made to the prototype checklist. Changes were made in graphic layout, and some items were rephrased. Observational data like late notice anaesthesia preparations, unlabeled and dispatched biopsy samples, and lack of hospital admission arrangements when needed led to addition of items on the checklist, as they were deemed critical for the optimal care process. Removed items concerned the checking of monitoring equipment and the availability of sedatives and antidotes. During observation, it was found that organizing these items in one systematic check performed by the technicians at the beginning of the day would be more efficient.

The final RADPASS checklist is divided into two parts: A (Planning and Preparation) and B (Procedure). The latter part is separated in checks just before starting a procedure (B1) and in items concerning the care immediately after completion of the procedure (B2). The checklist is reproduced as Table 1. In elective patients, part A was assessed the day before the procedure; in semielective patients, part A was completed on the day of the procedure.

**Table 1:**
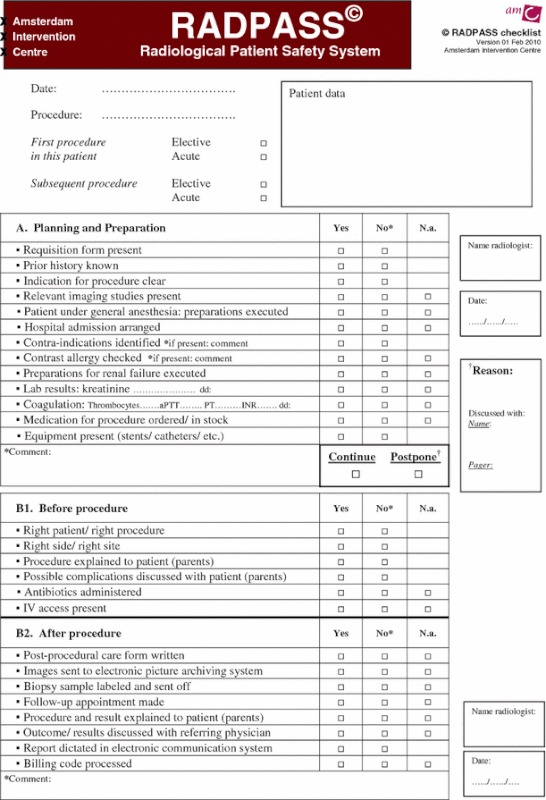
The RADiological Patient Safety System (RADPASS) checklist

### RADPASS Checking Process

All items, both on part A and part B, were checked by an interventional radiologist or a fellow or a resident under the supervision of a senior interventional radiologist. Use of anticoagulation medication was checked in advance by the referring physician, but even so, it was double-checked the day before the intervention by an interventional radiologist while completing the checklist. When aberrations were found, the referring physician was contacted and the possibility of continuing was discussed.

### Effect of RADPASS

In the preimplementation measurement, 94 procedures were observed, versus 101 procedures in the postimplementation measurement. Patient characteristics are listed in Table 2. In the postimplementation period, there were more semielective and fewer elective procedures included compared to the preimplementation period. The overall mean percentage of process deviations per procedure decreased from 24 % before implementation to 5 % after implementation (*p* < 0.001; Table 3). The improvement in processes was equal in semielective and elective procedures (data not shown). Before implementation, 10 % of included interventional procedures were postponed or canceled, whereas after implementation, no postponements or cancellations occurred (Table 3). Causes for postponement or cancellation during the preimplantation observations were missing patient or procedure information, absence of physician (IR or referring), or an uncorrected anticoagulative status.

**Table 2:** Patient characteristics

Characteristic	Before implementation (*n* = 94)	After implementation (*n* = 101)	*p*
Age (mean ± SD)	54.2 ± 18.7	59.0 ± 17.5	0.06
Male	44 (47 %)	54 (54 %)	0.32
Urgency			0.02
Semielective	8 (9 %)	21 (21 %)	
Elective	86 (92 %)	80 (79 %)	
Procedure			0.87
First procedure	56 (60 %)	59 (58 %)	
Subsequent procedure	38 (40 %)	42 (42 %)	
Type of procedure			0.45
Nonvascular			
Biliary drainage and liver procedures	18 (19 %)	22 (22 %)	
Imaging guided biopsy	16 (17 %)	20 (20 %)	
Drainage of fluid collection	14 (15 %)	12 (12 %)	
Tube change	6 (6 %)	4 (4 %)	
Radiofrequency ablation	1 (1 %)	1 (1 %)	
Vascular			
Percutaneous transluminal angioplasty	19 (20 %)	15 (15 %)	
Embolization	7 (7 %)	9 (9 %)	
Venous procedure	5 (5 %)	1 (1 %)	
Placement of central venous catheter	4 (4 %)	13 (13 %)	
Arteriovenous shunt intervention	2 (2 %)	1 (1 %)	
Placement of vena cava filter	2 (2 %)	3 (3 %)

**Table 3:** Process deviations and stopped procedures before and after implementation of RADPASS

Characteristic	Before implementation (*n* = 94)				After implementation (*n* = 101)				*p*
	Not applicable		Deviant items^a^		Not applicable		Deviant items^a^		
	*n*	(%)	*n*	(%)	*n*	(%)	*n*	(%)	
Preprocedural deviations from optimal process									
History known	0	(0)	18	(19)	0	(0)	1	(1)	
Indication discussed with referring physician	0	(0)	9	(10)	0	(0)	1	(1)	
Relevant imaging studies present	2	(2)	10	(11)	4	(4)	0	(0)	
Contraindications checked	0	(0)	25	(27)	0	(0)	2	(2)	
Contrast allergy checked	55	(59)	13	(33)	56	(55)	3	(7)	
Preparation for renal failure checked	65	(69)	19	(66)	72	(71)	2	(7)	
Lab results: creatinine checked	57	(61)	24	(65)	56	(55)	4	(9)	
Coagulation profile checked	18	(19)	37	(49)	16	(16)	7	(8)	
Medication for procedure ordered/in stock	47	(50)	14	(30)	51	(50)	3	(6)	
Equipment present (stents, catheters, etc.)	0	(0)	19	(20)	0	(0)	3	(3)	
Hospital admission arranged	47	(50)	2	(4)	52	(51)	0	(0)	
Total no. deviations before procedure			190				26		
Mean percentage				23 %				3 %	<0.001
Deviations from optimal process during procedure									
Right patient verified	0	(0)	0	(0)	0	(0)	0	(0)	
Right procedure/right side verified	0	(0)	3	(3)	0	(0)	0	(0)	
IV access present	22	(23)	6	(8)	35	(35)	0	(0)	
Availability of sedatives and antidotes checked	30	(32)	7	(11)	30	(30)	0	(0)	
Monitoring equipment checked	30	(32)	4	(6)	26	(26)	0	(0)	
Antibiotics administered	68	(72)	10	(39)	72	(71)	3	(10)	
Procedure explained to patient	3	(3)	4	(4)	0	(0)	0	(0)	
Complications discussed with patient	3	(3)	33	(36)	0	(0)	13	(13)	
Total no. deviations during procedure			67				16		
Mean percentage				12 %				2 %	<0.001
Postprocedural deviations from optimal process									<0.001
Postprocedural care form written ^b^	19	(20)	9	(12)	16	(16)	0	(0)	
Images sent to picture archiving and communication system ^b^	24	(26)	31	(44)	26	(26)	9	(12)	
Follow-up appointment made ^b^	63	(67)	16	(52)	82	(81)	1	(5)	
Procedure and result explained to patient (parents) ^b^	7	(7)	5	(6)	5	(5)	0	(0)	
Outcome/result discussed with the referring physician ^c^	42	(45)	28	(54)	63	(62)	3	(8)	
Report dictated in electronic reporting system ^c^	3	(3)	68	(75)	0	(0)	39	(39)	
Total no. deviations after procedure			157				52		
Mean percentage				39 %				13 %	<0.001
Total no. of deviations			414				94		
Overall mean percentage of deviations				24 %				5 %	<0.001
Stopped procedures									
Procedures postponed, *n* (%) ^d^			6	(6)				(0)	
Procedures canceled, *n* (%)^e^			3	(3)				(0)	

^a^Percentage of applicable items

^b^Item scored deviant when failed to complete within half an hour after completion of procedure

^c^Item scored deviant when failed to complete within an hour after completion of procedure

^d^As a result of missing information (*n* = 3), physician not present (*n* = 2), or coagulation not corrected

^e^As a result of missing information (*n* = 2) or coagulation not corrected

### Evaluation

After completion of the study, structured evaluation interviews were conducted with four staff interventional radiologists, four fellows, and three radiological technicians. Suggestions on workflow management were incorporated in the final version of RADPASS. Ten out of 11 interviewees considered the checklist user-friendly; 9 out of 11 would rather work with the checklist than without. All users agreed that the checklist improved patient safety awareness; 7 out of 11 users agreed that the checklist improved efficiency.

## Discussion

The need for more standardization to improve patient safety and quality of care is increasingly being recognized in IR. The use of the RADPASS checklist, a validated comprehensive patient safety checklist in IR, was associated with a decrease in process deviations per procedure from 24 % before implementation to 5 % after implementation. The proportion of postponed and canceled procedures decreased from 10 to 0 %. After a 6-month period of use, 91 % of users found the checklist user-friendly, and all users believed that patient safety had increased by using RADPASS.

For coagulation profile, creatinine levels, and renal failure precautionary measurements, the percentage of process deviations was relatively high. These parameters might have been checked in some way at some time, but in the process, there had been no evident checking moment of these items, leaving them to be verified only by chance.

The checklist is intended to be used as a framework for the radiological intervention. It constitutes safety checks while preparing and finishing an intervention. At the same time, it aspires to improve time efficiency by enforcing timely preparation for all procedures and thereby identifying difficulties in advance.

There is no abundance of publications about adverse events or errors in IR. One article described the data from 10 years of morbidity and mortality conferences in a pediatric IR service [21]. Although the reported incidents were not described in detail, they were divided into categories; the majority consisted of procedure-, patient-, or process-related incidents. Almost half of all improvement recommendations resulting from the morbidity and mortality conferences concerned process improvements.

Although a comprehensive safety checklist like RADPASS has not been described before, there have been a number of publications about checklists in IR. Two publications have described application of the “Universal Protocol for Preventing Wrong Site, Wrong Procedure, Wrong Person Surgery” to the practice of IR [4, 18]. In these publications, a number of issues were identified that are unique to IR and required adaptation of the universal protocol, including, among others, the positioning of the patient relative to the imaging field and the use of intraprocedural imaging to determine the site of the procedure. Both factors pose challenges for reliably marking the intervention site, and the universal protocol has been adapted to accommodate these factors. However, the universal protocol focuses mostly on preventing wrong side/site interventions, whereas the present checklist aims to improve the entire process surrounding radiological interventions. Another article in the “IR Safety Rounds” described the development of a very short postprocedural checklist to ensure dialysis catheter caps were not forgotten [22].

There are a number of possible limitations to this study. Firstly, the effect of the checklist was assessed in a before–after measurement. Thus, it is possible that the observed changes were not entirely caused by the implementation of RADPASS but rather affected by changes in time or case mix. However, both measurements were conducted within a 1-year time frame; no other changes in policy occurred in the department of IR during this year. In addition, there were no significant differences in case mix, other than less elective procedures in the postimplementation period making the observed effect even stronger. Secondly, the results may have been subject to a Hawthorne effect. During the observations, interventional radiologists, fellows, and laboratory technologists might have been influenced by the fact that they were being observed. This might have enhanced the amount of usual preparation, leading to an underestimation of the number of process deviations in usual practice. However, this limitation applies to both before and after measurement and thus is unlikely to have influenced the difference we found, particularly because the checklist was introduced as a quality project and the measurements of deviations were never elucidated to the participating IR personnel. Thirdly, only few observations were performed. Whether the beneficial effect of RADPASS will be sustained in the long run remains to be seen. Finally, process deviations represent a surrogate end point: in the end, the goal of RADPASS is to improve patient safety by reducing adverse events related to IR. Clinical data of later occurring complications were insufficient, mainly because of the distribution of patients treated from within the hospital or as a referral center for other hospitals. This interferes with patient follow-up and hinders an accurate and reliable registration. Therefore, analysis on the effect of the RADPASS checklist on the number of complications was not possible.

Nonetheless, it is likely that a reduction in process deviations will eventually lead to a reduction in adverse outcomes. This assumption will need to be tested in future studies.

Strengths of this study include the novelty of the concept, the large variety of procedures the checklist was tested in, and the use of different sources of information (literature study, expert panel, observation techniques, evaluation interviews) to develop and study the checklist [23].

Checklists increase the safety and reliability of care and reduce the risk of errors occurring. In IR, the use of checklists is gradually being introduced. The RADPASS checklist covers all stages of the pathway for IR procedures (planning, preparation, and day-of-treatment and postprocedural care). It is a generic checklist that can be used in all settings and includes three Joint Commission on Accreditation in Healthcare Organizations safety goals: improving the accuracy of patient identification, improving communication between caregivers, and eliminating wrong-site, wrong-patient, and wrong-procedure procedures [24]. The use of this checklist led to a significant decrease in process deviations and procedure postponements.

## References

[1] de Vries EN, Ramrattan MA, Smorenburg SM (2008). The incidence and nature of in-hospital adverse events: a systematic review. Qual Saf Health Care.

[2] Christian CK, Gustafson ML, Roth EM (2006). A prospective study of patient safety in the operating room. Surgery.

[3] Dankelman J, Grimbergen CA (2005). Systems approach to reduce errors in surgery. Surg Endosc.

[4] Angle JF, Nemcek AA, Cohen AM (2008). Quality improvement guidelines for preventing wrong site, wrong procedure, and wrong person errors: application of the joint commission “Universal Protocol for Preventing Wrong Site, Wrong Procedure, Wrong Person Surgery” to the practice of interventional radiology. J Vasc Interv Radiol.

[5] Duncan JR (2008). Strategies for improving safety and quality in interventional radiology. J Vasc Interv Radiol.

[6] Jacobs B, Duncan JR (2009). Improving quality and patient safety by minimizing unnecessary variation. J Vasc Interv Radiol.

[7] Miller DL (2007). Safety in interventional radiology. J Vasc Interv Radiol.

[8] The aviation safety system. Federal Aviation Administration (2006) http://www.faa.gov/data_research/safety/

[9] Common sense at work. Occupational Safety and Health Administration (2006) http://www.osha.gov/oshstats/index.html

[10] Kohn LT (1999) To err is human: building a safer health care system. Institute of Medicine. http://books.nap.edu/openbook.php?isbn=0309068371 11995167

[11] Haynes AB, Weiser TG, Berry WR (2009). A surgical safety checklist to reduce morbidity and mortality in a global population. N Engl J Med.

[12] de Vries EN, Prins HA, Crolla RMPH (2010). Effect of a comprehensive surgical safety system on patient outcomes. N Engl J Med.

[13] Stecker MS (2007). Root cause analysis. J Vasc Interv Radiol.

[14] Lee MJ, Fanelli F, Haage P (2012). Patient safety in interventional radiology: a CIRSE IR checklist. Cardiovasc Intervent Radiol.

[15] de Vries EN, Hollmann MW, Smorenburg SM (2009). Development and validation of the SURgical PAtient Safety System (SURPASS) checklist. Qual Saf Health Care.

[16] Borgstede JP, Zinninger MD (2004). Radiology and patient safety. Acad Radiol.

[17] Cardella JF, Kundu S, Miller DL (2009). Society of Interventional Radiology clinical practice guidelines. J Vasc Interv Radiol.

[18] Knight F, Galvin R, Davoren M (2006). The evolution of universal protocol in interventional radiology. J Radiol Nurs.

[19] Sridhar S, Duncan JR (2008). Strategies for choosing process improvement projects. J Vasc Interv Radiol.

[20] Thrall JH (2004). Quality and safety revolution in health care. Radiology.

[21] Tuong B, Shnitzer Z, Pehora C (2009). The experience of conducting mortality and morbidity reviews in a pediatric interventional radiology service: a retrospective study. J Vasc Interv Radiol.

[22] Stecker MS, McNamara D (2007). IR safety rounds: forgotten dialysis catheter caps. J Vasc Interv Radiol.

[23] Brown C, Hofer T, Johal A (2008). An epistemology of patient safety research: a framework for study design and interpretation. Part 4 One size does not fit all. Qual Saf Health Care.

[24] Anonymous (2008) The Joint Commission announces the 2009 national patient safety goals and requirements Jt Comm Perspect 28(1):11–15 18795721

